# Hypervascular tumor volume estimated by comparison to a large-scale cerebral blood volume radiographic atlas predicts survival in recurrent glioblastoma treated with bevacizumab

**DOI:** 10.1186/s40644-014-0031-z

**Published:** 2014-11-14

**Authors:** Kevin Leu, Dieter R Enzmann, Davis C Woodworth, Robert J Harris, Anh N Tran, Albert Lai, Phioanh L Nghiemphu, Whitney B Pope, Timothy F Cloughesy, Benjamin M Ellingson

**Affiliations:** 1 Department of Radiological Sciences, David Geffen School of Medicine, University of California Los Angeles, Los Angeles, USA; 2 Department of Bioengineering, Henry Samueli School of Engineering and Applied Science, University of California Los Angeles, Los Angeles, USA; 3 Department of Biomedical Physics, David Geffen School of Medicine, University of California Los Angeles, Los Angeles, USA; 4 Department of Neurology, David Geffen School of Medicine, University of California Los Angeles, Los Angeles, USA; 5 UCLA Brain Tumor Imaging Laboratory (BTIL), Center for Computer Vision and Imaging Biomarkers (CVIB), Department of Radiological Sciences, David Geffen School of Medicine, University of California, 924 Westwood Blvd., Suite 615, Los Angeles 90024, CA, USA

**Keywords:** CBV, Recurrent GBM, Population map, Radiographic atlas, Bevacizumab

## Abstract

**Background:**

Dynamic susceptibility contrast (DSC)-MRI is a well-established perfusion MR imaging technique for estimating relative cerebral blood volume (CBV) in primary brain tumors; however, tumors localized to regions with naturally elevated perfusion, including cortical tissue and common vascular territories, make evaluation of tumor vascularity difficult to assess. In the current study, we have constructed a large-scale radiographic atlas of CBV to assess treatment response to bevacizumab in individual patients with recurrent glioblastoma.

**Methods:**

Z-score normalized CBV maps were registered to stereotactic atlas space in 450 patients with brain tumors. A CBV atlas was created by calculating the voxel-wise mean and variability in CBV. MRI and CBV maps from 32 recurrent glioblastoma patients were then obtained prior to and following treatment with bevacizumab, registered to and compared with the CBV atlas. The volume of tumor tissue with elevated CBV, percentage of enhancing tumor with elevated CBV, and the mean and maximum change in normalized CBV intensity relative to the atlas were computed.

**Results:**

Voxel-wise comparison of individual patient CBV maps to the atlas allowed delineation of elevated tumor perfusion from artery and normal cortical tissue. An atlas-defined hypervascular tumor blood volume greater than 2.35 cc prior to treatment, 0.14 cc after treatment, and a decrease in atlas-defined hypervascular tumor volume less than 80% following treatment were characteristic of a shorter PFS and OS. Traditional measures of CBV were not predictive of PFS or OS.

**Conclusions:**

This study highlights the advantages of large-scale population maps to identify abnormal biological tissues.

## Background

Malignant primary brain tumors are among the most aggressive and devastating cancers, frequently leading to profound disabilities and death. Glioblastoma multiforme (GBM), the most aggressive form of malignant glioma, carries a particularly poor patient prognosis [1]. Standard initial therapy for GBM consists of maximal surgical resection followed by aggressive radiochemotherapy [2],[3] and yields a median survival of between 12 and 15 months [2],[3].

Malignant gliomas thrive by co-opting pre-existing vasculature and inducing formation of new blood vessels [4]–[6]. This finding has ushered in the use of anti-angiogenic therapies to normalize the tumor vasculature for the purposes of increased penetration of chemotherapeutics or starving the tumor of nutrients. Bevacizumab, a humanized monoclonal antibody for VEGF, is the only anti-angiogenic agent FDA-approved for use in recurrent GBM. The potential alterations of anti-angiogenic therapies on tumor vasculature has led to the interest in surrogate biomarkers for evaluating therapeutic response [7]. Dynamic susceptibility contrast (DSC)-MRI, a specific perfusion magnetic resonance imaging (MRI) technique that can be used to estimate cerebral blood volume (CBV) or blood flow (CBF), has been shown to predict tumor grade [8]–[10] and predict survival in low-grade gliomas [11]. Preliminary results in anti-angiogenic therapy, however, have not been particularly fruitful and, at times, appear counter-intuitive [12].

A wide variability in MR scanner hardware and acquisition parameters leading to variability in CBV measurements are likely a significant factor contributing to the lack of correlation observed between perfusion MRI and response to anti-angiogenic agents. Therefore, in the present work, we chose to utilize a population-based CBV atlas derived from the normal tissue in 450 CBV maps from 450 different patients collected under a variety of scan protocols in order to maximize the specificity for identify hypervascular tissue in GBM patients. In this way, the variability in CBV due to different hardware and sequence parameters are captured within the CBV atlas, requiring a high threshold of CBV within tumor for classification as abnormal. Using this approach, we investigated the relationship between CBV abnormalities relative to this atlas and survival in 32 patients with recurrent glioblastoma treated with bevacizumab. Specifically, we measured the volume of hypervascularity, percentage of hypervascular tumor within areas of contrast enhancement, and mean CBV of hypervascular tissue.

## Methods

### Patient population

#### CBV Atlas

A total of 450 patients with DSC-MRI were retrospectively selected from our neuro-oncology database. All patients gave appropriate institutional review board approved informed consent to have their information stored in our database (UCLA IRB #10-000655; Reviewed by Medical IRB Panel 2). All image acquisition and processing was performed according to appropriate Health Insurance Portability and Accountability Act (HIPAA) regulations. All patients included in the atlas had brain tumors and were not selected based on any specific histological or treatment criteria. In particular, a total of 83 WHO II, 136 WHO III, and 231 WHO IV tumors were included, of which 43 patients were not undergoing any systemic therapy, 20 patients had undergone previous external beam radiotherapy only, 20 patients underwent previous chemotherapy only, and 367 patients had previously undergone both external beam radiation therapy and chemotherapy at the time of DSC-MRI acquisition. No patients were treated with anti-angiogenic therapies, immunotherapies, or other experimental therapies at the time of perfusion MRI. Additionally, a total of 236 patients did not have any previous tumor progression, 163 patients had one tumor progression, and 51 patients had two or more tumor recurrences prior to perfusion MRI acquisition. The rationale for including a wide range of patients and treatments was to maximize the specificity for detecting hypervascular tissue within the primary tumor region in subsequent comparisons with brain tumor patients. After DSC-MRI data was obtained for atlas patients, regions of tumor defined by T2/FLAIR hyperintensity were removed during atlas creation to ensure the atlas consisted of only normal-appearing brain tissue. (Of course, there is the possibility of invading tumor beyond T2/FLAIR hyperintense regions; however, these regions are not likely to contain tumor with significant angiogenesis). The dates of acquisition of perfusion MR scans included in the atlas ranged from July 2010 to August 2012.

#### Bevacizumab therapy

A total of 32 patients with recurrent GBM treated with bevaciumab, separate from the patients used in the atlas, were chosen from our neuro-oncology database. Patients included in this analysis were not included in calculation of the CBV atlas and were also selected from scans acquired between October 2010 and July 2013. For each of these patients, one DSC-MRI scan within one month prior to treatment with bevacizumab (pre-treatment) and within 3 months following the first dose of bevacizumab (post-treatment) were used for analysis. Additionally, all patients treated with bevacizumab at recurrence were at least one month after completion of radiotherapy to limit contributions of pseudoprogression. Patients were also excluded if DSC-MRI did not have full coverage of the contrast-enhancing lesion on post-contrast T1-weighted images.

### Magnetic resonance imaging

DSC-MR images and the post-contrast T1-weighted images were acquired using standard pulse sequences on either 1.5 T MR (Siemens Avanto, Siemens Sonata, Siemens Symphony, Siemens Magnetom Vision, Siemens Healthcare; GE Genesis, GE Signa Excite, GE Signa HDx, GE Medical Systems; Philips Intera, Philips Medical Systems) or 3.0 T MR (Siemens Trio, Siemens Healthcare). A 0.025 mmol/kg pre-load dose of gadolinium contrast agent was administered prior to DSC-MRI to diminish T1 relaxation effects of contrast agent extravasation [10],[13],[14]. A 3-5 cc/sec bolus of either gadopentetate dimeglumine (Gd-DTPA; Magenvist, Bayer Schering Pharma, Leverkusen, Germany), administered at a dose of 10-20 cc (0.075 mmol/kg), or gadobenate dimeglumine (Gd-BOPTA; Multihance, Bracco Diagnostics, Princeton, NJ), administered at a dose of 9-20 cc (0.075 mmol/kg), was used in the acquisition of DSC as well as the subsequent post-contrast T1-weighted images (total of 0.01 mmol/kg). DSC-MRI scan parameters ranged from 23-50 ms for echo times (TE), 1250-1400 ms for repetition times (TR), 30-35 for flip angles (FA), 40-90 repetitions, 4-7 mm for slice thickness with interslice gap from 0-1.5 mm, 6 – 20 for number of slices, and 80 × 96 to 128 × 128 for matrix sizes.

### DSC-MRI data analysis

Parametric maps were calculated using the commercially available post-processing software (IB Neuro v2.0; Imaging Biometrics, Elm Grove, WI) from the MR perfusion images. The analysis of the DSC-MRI images consisted of the following steps: 1) truncation of the first five time points, at which point the MR signal will reach its steady state; 2) conversion of the DSC-MRI time series to a concentration-time curve based on the T2* relaxivity of the contrast agent, using the pre-bolus signal intensity as a correction factor; 3) estimation of CBV on a voxel-wise basis through a 120-point trapezoidal integration method, with correction for leakage [10]; and 4) standardization of the CBV grayscale values [15].

### Initial affine registration

All images (450 CBVs for the atlas, along with 64 CBVs, which consisted of 32 pre-treatment CBVs, 32 post-treatment CBVs, and corresponding T1 post-contrast images from 32 patients, for survival analysis) for each patient were registered to a 1.0-mm isotropic brain atlas (Montreal Neurological Institute 152) by using a mutual information algorithm and a 12 degrees of freedom transformation through the Functional MR Imaging of the Brain Software Library (http://www.fmrib.ox.ac.uk/fsl/). Adequate alignment was determined ensuring less than ±5 degrees rotation and less than ±2.5 mm translation of the brain defined by specific anatomic landmarks (e.g. AC-PC line, edge of brain, alignment of ventricles, etc.). This was followed by visual inspection by two independent raters (B.M.E., more than 5 years of experience, and WBP, more than 10 years of experience) to verify adequate alignment.

### Regions of interest

The regions of interest (ROI) were created using custom scripts in Analysis of Functional NeuroImages (AFNI) software (http://afni.nimh.nih.gov/afni). The process involved 1) manually defining the relative region of tumor occurrence, 2) thresholding the post-contrast T1-weighted images, both pre-treatment and post-treatment, in the 32 GBM patients, and 3) manually editing the resulting masks to exclude necrotic areas. ROIs were drawn on all slices that contained enhancing tumor. Correct segmentation was verified independently by two investigators (K.L, B.M.E.).

### Definition of disease progression

Progression was defined prospectively by the treating neuro-oncologists if subsequent scans showed an increase in imaging-evaluable tumor (≥25% increase in the sum of enhancing lesions, new enhancing lesions >1 cm^2^, an unequivocal qualitative increase in nonenhancing tumor, or an unequivocal new area of noncontrast enhancing tumor). Change in steroid dosage was taken into consideration while defining progression. More specifically, patients were required to have stable or decreasing contrast agent dose before partial or complete response could be determined. Additionally, patients requiring increased dosage of steroids in order to maintain neurologic function, even in the absence of worsening on anatomical images, were considered to be stable, but required early reevaluation. Patients who experienced significant neurologic decline were also declared to have progressed at the time of irreversible decline. Progression-free survival (PFS) was therefore defined as being the number of days between the start of bevacizumab and declared progression. Overall survival (OS) was defined as the number of days between the start of bevacizumab treatment to death. For all assessments that required the use of the post-treatment scan, landmark analyses were performed. Landmark survival was defined as the difference between 1) the original PFS or OS values and 2) the number of days between the time of the post-treatment scan and progression.

### Traditional cerebral blood volume measurements

Standardized CBV maps were used for the 32 GBM patients on bevacizumab as a traditional approach to quantifying CBV. Mean and maximum CBV was measured within the tumor ROIs mentioned above. Statistical analyses were performed in the same manner as those performed with atlas-defined perfusion parameters.

### CBV atlas-defined perfusion parameters

The atlas was created using custom bash scripts and AFNI. CBV data for each patient was normalized by custom c-code and bash scripts, courtesy of the National Institutes of Mental Health Magnetoencephallography Core Facility (3dNormalize; NIMH MEG Core, Bethesda, MD; kurage.nimh.nih.gov/meglab/Med/3dNormalize). Briefly, the standardized CBV maps for each patient were subtracted by the mean CBV of the whole brain and divided by the standard deviation of CBV throughout the brain to obtain a resultant z-score normalized map of CBV measurements. Whole brain CBV measures were obtained by first segmenting the brain using the brain extraction tool (BET) then calculating both the mean and standard deviation of CBV throughout the entire brain mask. This method of normalization has previously been shown to reduce variability in CBV measurments in the same patient over time, across scanner platforms, and across acquisition protocols [16]. After registration to MNI space, the average z-score normalized CBV for each image voxel was calculated across all 450 patients, excluding areas of tumor as defined by T2/FLAIR hyperintense regions, leaving only regions of relatively normal tissue for formation of the atlas. Exclusion of these regions was chosen based on the large number of non-enhancing, low-grade gliomas included in the atlas as well as recent evidence from Jain *et al.*[17] demonstrating that CBV within these regions are altered in high-grade gliomas.

The same z-score normalization procedure was performed on each of the individual recurrent glioblastoma patients treated with bevacizumab. The individual z-score normalized CBV maps were subsequently subtracted from the CBV atlas on a voxel-by-voxel basis. Atlas-defined perfusion parameters included: 1) the volume of atlas-defined elevated tissue within T1 post-contrast lesions; 2) the percentage of the tumor with elevated CBV; and 3) the mean value of atlas-corrected elevated CBV tissue within the same ROI.

### Statistical analysis

Patient stratification was optimized according to level of significance (*p-value*) on univariate Cox proportional hazards regression analysis with the parameter of interest and either PFS or OS, using the “*coxphfit*” function in the MATLAB Statistics Toolbox. The thresholds established in this regression model were used to create Kaplan-Meier survival curves and their subsequent log-rank analyses. To avoid having extremely skewed groups (e.g., 3 patients in one group versus 29 in the other), the number of patients either above or below the cutoff was at minimum 1/3 of the patient sample. A multivariate Cox proportional regression model was subsequently performed on all significant parameters according to the log-rank analysis using MATLAB. Predictors included age, tumor volume, and the parameter being examined, while the observed response was either OS or PFS. Tumor volume in the regression matched the time point of the scan. For instance, if the parameter was based on atlas defined pre-treatment elevated CBV volume, then “tumor volume” was the volume of enhancing tumor in the pre-treatment scan. For the top-performing parameters, an ROC curve analysis was performed on one of the following: 6-month OS, 5-month landmark OS, 3-month PFS, or 2-month landmark PFS at the determined threshold, depending on the time point at which the parameter was measured and what clinical endpoint was used.

## Results

**Figure 1:**
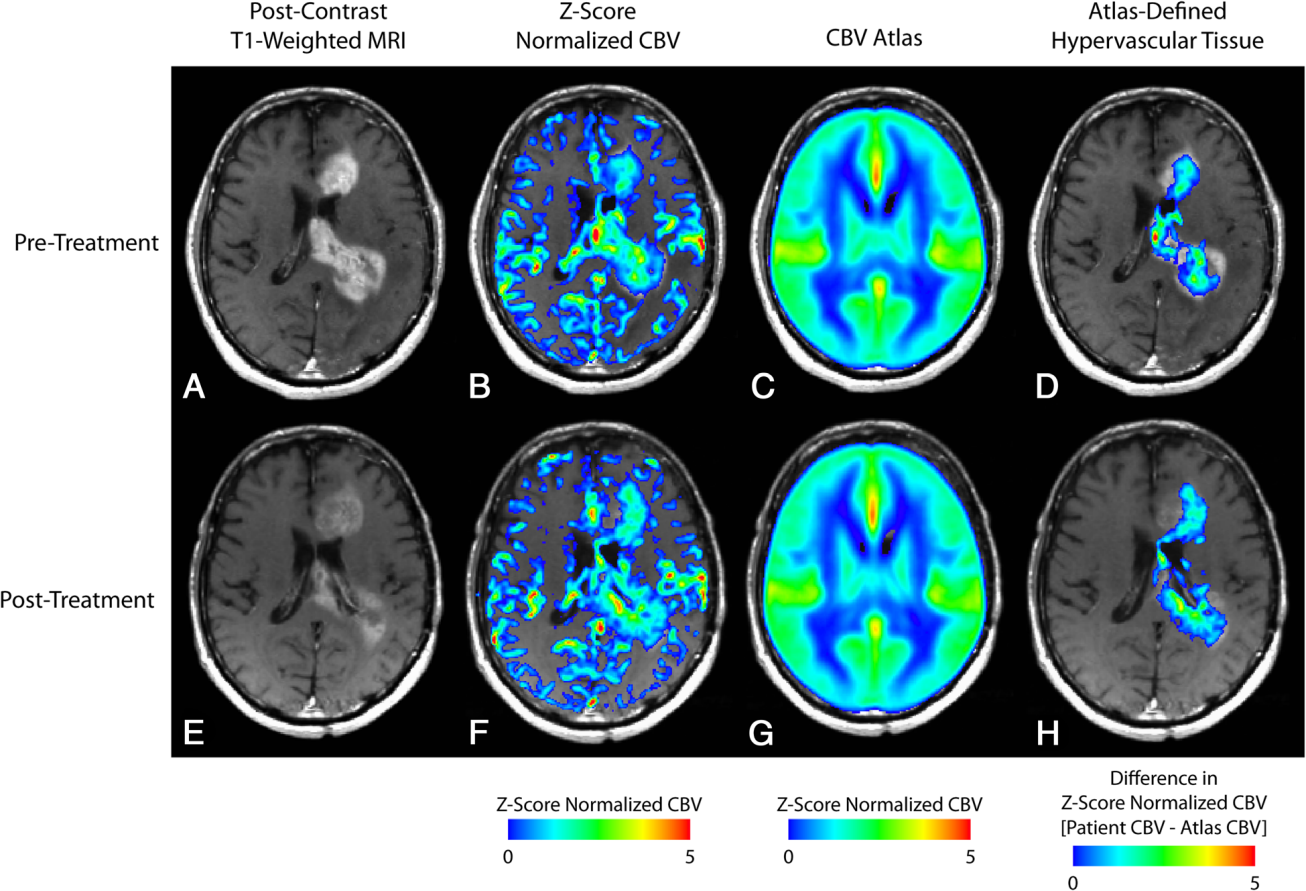
**Non-responder to bevacizumab (PFS =36 days; OS =62 days). A)** Pre-treatment, post-contrast T1-weighted image. **B)** Pre-treatment, Z-score normalized CBV. **C)** CBV atlas. **D)** Pre-treatment, atlas-defined hypervascular tumor tissue showing a large extent of abnormal vasculature prior to therapy. **E)** Post-treatment, post-contrast T1-weighted image. **F)** Z-score normalized CBV. **G)** CBV atlas. **H)** Post-treatment, atlas-defined hypervascular tumor tissue showing a large residual volume of abnormal vasculature after administration of bevacizumab.

**Figure 2:**
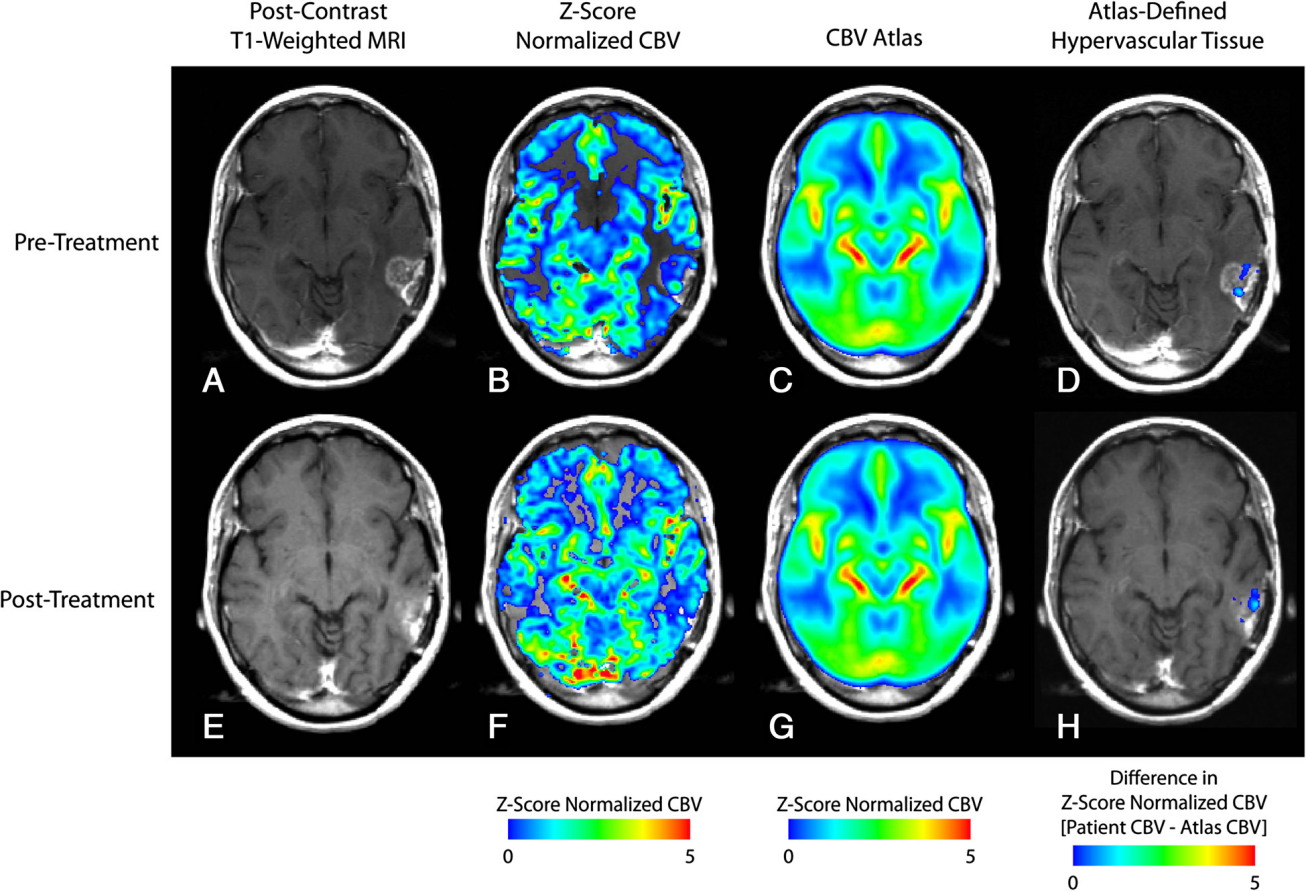
**Responder to bevaciuzumab (PFS =114 days; OS =486 days). A)** Pre-treatment, post-contrast T1-weighted image. **B)** Pre-treatment, Z-score normalized CBV. **C)** CBV atlas. **D)** Pre-treatment, atlas-defined hypervascular tumor tissue showing a relatively small extent of abnormal vasculature prior to therapy. **E)** Post-treatment, post-contrast T1-weighted image. **F)** Z-score normalized CBV. **G)** CBV atlas. **H)** Atlas-defined hypervascular tumor tissue showing a small residual volume of abnormal vasculature after administration of bevacizumab.

The resulting CBV atlas is comprised of the composite, average z-score normalized CBV values across a total of 450 patients (Figures 1C,G and 2C,G). The resulting atlas indicates the ventricles and periventricular areas have low values of CBV compared to the rest of the brain. As expected, the CBV atlas also demonstrated areas of high CBV in and adjacent to cortical tissue as well as along the sagittal midline.

Next, we investigated the utility of the CBV atlas in defining hypervascular tissue in 32 patients with recurrent glioblastoma treated with bevacizumab. In particular, we examined the volume of atlas-defined hypervascular tissue, the percentage of enhancing tumor with elevated CBV, and the mean value of abnormally high CBV tissue before and after bevacizumab to determine whether these measurements of angiogenesis could predict PFS and OS. In general, patients with a high volume of hypervascular tissue prior to or after administration of bevacizumab appeared to have a shorter PFS and OS compared with patients exhibiting a lower volume of hypervascular tissue. Figure 1 shows a patient that did not respond to bevacizumab therapy, as indicated by a short PFS of 36 days and OS of 62 days. Atlas-defined hypervascular volume was relatively high and unchanged for this patient, suggesting that the extent of tissue with elevated tumor blood volume was not altered as a result of treatment. Figure 2 shows a patient with a relatively long PFS and OS (114 and 486 days, respectively) and a very small volume of hypervascular tissue both before and after bevacizumab therapy, suggesting patients with a small volume of hypervascular tumor may have a favorable response to bevacizumab upon recurrence.

**Figure 3:**
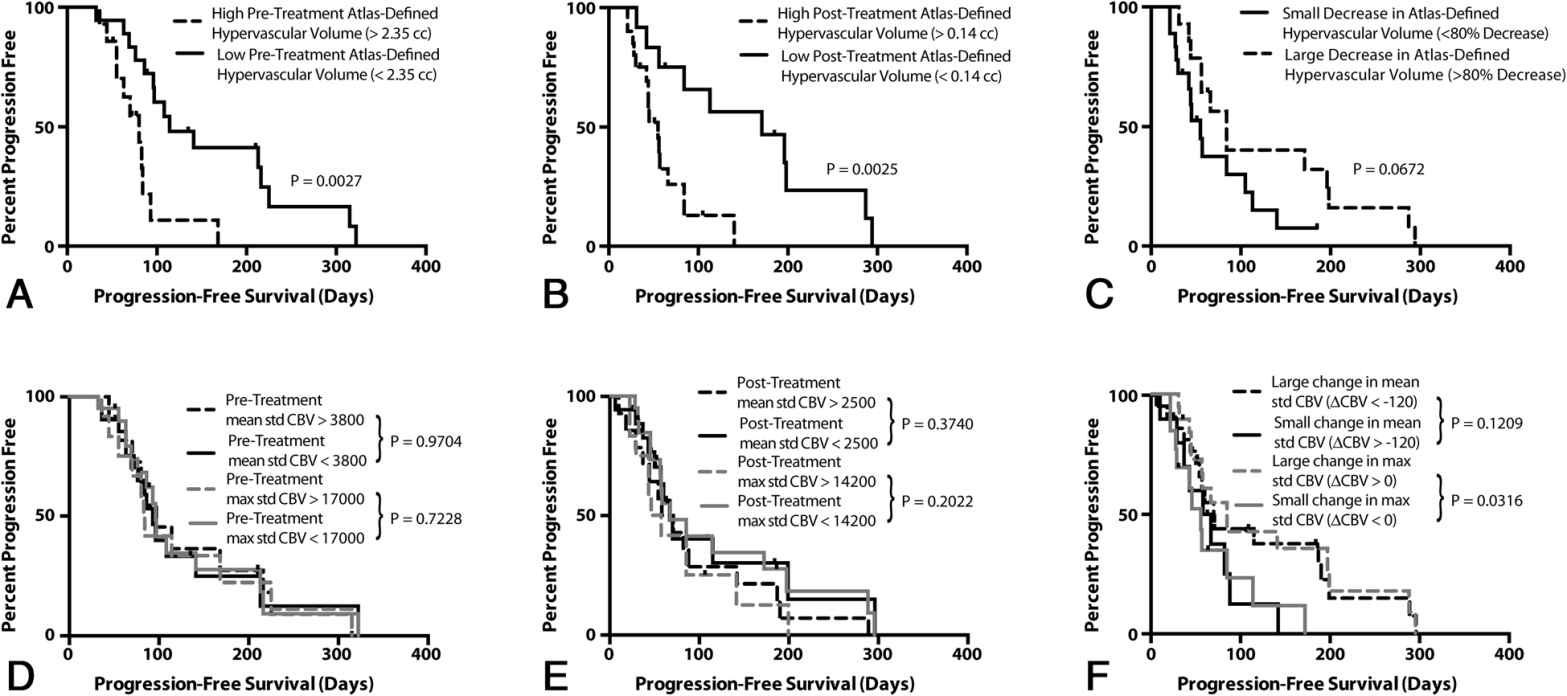
**Relationship between perfusion parameters and progression-free survival (PFS). A)** Pre-treatment, atlas-defined hypervascular volume (Log-rank, P = 0.0027). **B)** Post-treatment, atlas-defined hypervascular volume (Log-rank, P = 0.0025). **C)** Change in atlas-defined hypervascular volume (Log-rank, P = 0.0672). **D)** Pre-treatment, mean standardized CBV (black lines; Log-rank, P = 0.9704) and maximum standardized CBV (gray lines; Log-rank, P = 0.7228). **E)** Post-treatment, mean standardized CBV (black lines; Log-rank, P = 0.3740) and maximum standardized CBV (gray lines; Log-rank, P = 0.2022). **F)** Change in mean standardized CBV (black lines; Log-rank, P = 0.1209) and maximum standardized CBV (gray lines; Log-rank, P = 0.0316).

Univariate results suggested that many perfusion parameters were predictive of PFS (Table 1). When examining the perfusion in pre-treatment setting, patients with an atlas-defined hypervascular volume greater than 2.35 cc had a significantly shorter PFS compared with patients exhibiting a lower hypervascular volume (Figure 3A, P = 0.0027). No other pre-treatment perfusion parameters showed significant stratification of PFS (Table 1), including traditional measures of mean standardized CBV (Figure 3D*, P = 0.9704*) and maximum standardized CBV *(*Figure 3D*, P = 0.7228)*. Similar to trends in pre-treatment perfusion, patients with a higher post-treatment atlas-defined hypervascular volume (>0.14 cc) had a significantly shorter PFS (Figure 3B*, P = 0.0025*). Similarly, no other post-treatment perfusion parameters showed stratification of PFS, including traditional measures of standardized mean CBV (Figure 3E*, P = 0.3740*) and maximum standardized CBV (*P = 0.2022*). Examination of the change in perfusion parameters after administration of bevacizumab indicated a small change in atlas-defined hypervascular volume trended toward a shorter PFS compared with patients showing a large change in hypervascular volume (Figure 3C*, P = 0.0672*). The more traditional measure, change in maximum standardized CBV between pre- and post-treatment scans, more successfully stratified PFS in this instance (Figure 3F*, P = 0.0316*). Multivariate Cox regression analysis indicated that pre-treatment, atlas-defined hypervascular tumor volume was the strongest predictor of PFS when age and contrast-enhanced tumor volume were also considered (Table 2*; P = 0.027, HR = 3.64*).

**Table 1:** Univariate analysis for PFS and OS

				**PFS**	**OS**
**Perfusion parameter**			**Threshold**	**[P-value]**	**[P-value]**
Atlas-defined perfusion parameters					
	Pre-Treatment				
		Hypervascular Volume	2.35 cc	0.0027**	0.0654
		Hypervascular Volume Fraction	9%	0.8236	0.0546
		Mean Z-Score Value	0.575	0.3521	0.1771
	Post-Treatment				
		Hypervascular Volume	0.14 cc	0.0025**	0.0304*
		Hypervascular Volume Fraction	8.9%	0.1320	0.0242
		Mean Z-Score Value	0.5	0.1023	0.0091**
	Change				
		Change in Volume	1 cc	0.2061	0.0563
		Percentage Change in Volume	80%	0.0672	0.0483*
		Change in Mean Z-Score	0.08	0.7764	0.0497*
Traditional perfusion parameters					
Mean rCBV [a.u.]					
	Pre-Treatment				
		Mean Standardized CBV	3800	0.9704	0.0872
	Post-Treatment				
		Mean Standardized CBV	2500	0.374	0.1499
	Change				
		Change in Mean Standardized CBV	120	0.1209	0.3475
Max rCBV [a.u.]					
	Pre-Treatment				
		Max Standardized CBV	17000	0.7228	0.0555
	Post-Treatment				
		Max Standardized CBV	14200	0.2022	0.0823
	Change				
		Change in Max Standardized CBV	0	0.0316*	0.6288

* = P < 0.05, ** = P < 0.01.

**Table 2:** Multivariate Cox regression for PFS and OS

**Multivariate Cox regression**	**Hazard ratio**	**Std error**	**95% CI**	**P-value**
**PFS**				
Age	0.98	0.02	0.94, 1.02	0.281
Pre-treatment enhancing volume (cc)	1.01	0.01	0.98, 1.03	0.665
Pre-treatment atlas-defined hypervasc. vol. (cc)	3.64	0.58	1.16, 11.41	0.027*
**OS**				
Age	1.04	0.03	0.98,1.10	0.195
Post-treatment enhancing volume (cc)	1.05	0.03	1.0,111	0.060
Post-treatment atlas-defined hypervasc. vol. (cc)	6.15	0.73	1.46,25.87	0.013*

*= P < 0.05.

**Figure 4:**
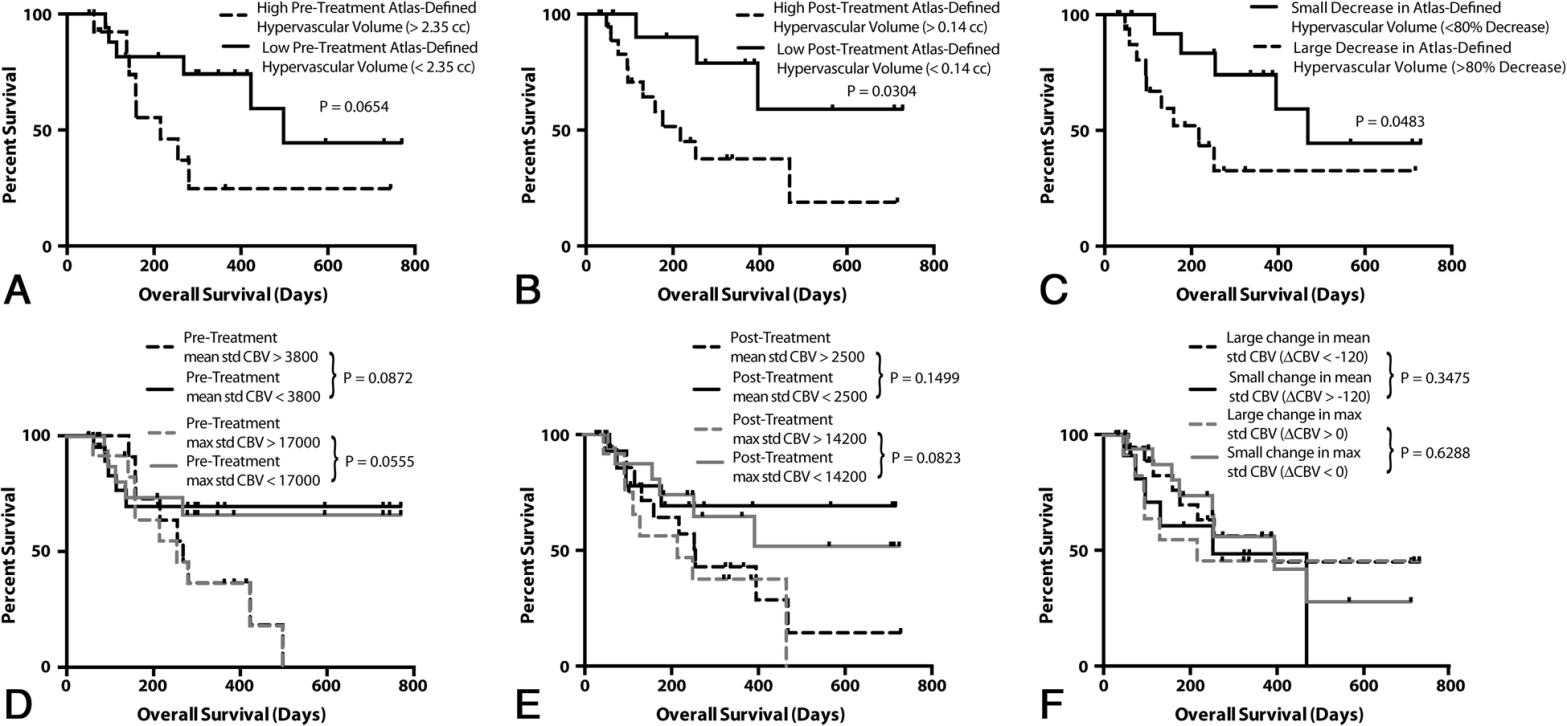
**Relationship between perfusion parameters and overall survival (OS). A)** Pre-treatment, atlas-defined hypervascular volume (Log-rank, P = 0.0654). **B)** Post-treatment, atlas-defined hypervascular volume (Log-rank, P = 0.0304). **C)** Change in atlas-defined hypervascular volume (Log-rank, P = 0.0483). **D)** Pre-treatment, mean standardized CBV (black lines; Log-rank, P = 0.0872) and maximum standardized CBV (gray lines; Log-rank, P = 0.0555). **E)** Post-treatment, mean standardized CBV (black lines; Log-rank, P = 0.1499) and maximum standardized CBV (gray lines; Log-rank, P = 0.0823). **F)** Change in mean standardized CBV (black lines; Log-rank, P = 0.3475) and maximum standardized CBV (gray lines; Log-rank, P = 0.6288).

Univariate analysis indicated that a large number of perfusion parameters were predictive of OS (Table 1). Similar to PFS, a high atlas-defined hypervascular tumor volume prior to bevacizumab therapy trended toward significantly shorter OS (Figure 4A*, P = 0.0654*), while traditional mean standardized CBV did not show as clear separation between groups (Figure 4D*, P = 0.0872*), nor did maximum standardized CBV (Figure 4*, P = 0.0555)*. High atlas-defined hypervascular tumor volume following bevacizumab therapy was also associated with a shorter OS (Figure 4B, *P = 0.0304*), while neither mean standardized CBV nor maximum standardized CBV was (Figure 4E*, P = 0.1499 and P = 0.0823, respectively*). Lastly, a decrease in atlas-defined hypervascular tumor volume of more than 80% was associated with a significantly longer OS compared with patients showing a change below this threshold (Figure 4C*, P = 0.0483*). Other atlas-defined perfusion parameters also showed significant associations with OS (Table 1). Traditional measures of change in mean and maximum standardized CBV again did not show an association with OS (Figure 4F, P = 0.3475 and P = 0.6288, respectively). Multivariate Cox regression analysis indicated that post-treatment, atlas-defined hypervascular tumor volume was the strongest predictor of OS when age and contrast-enhanced tumor volume were included as covariates (Table 2*; P = 0.013, HR = 6.15*).

## Discussion

In the current work we have effectively established a new method of delineating regions of “abnormal” CBV by leveraging perfusion information from a population map based on 450 tumor patients. This atlas-based approach is favorable compared to other approaches used in defining abnormal CBV in that it is both unbiased in terms of the choice of CBV that defines abnormal tissue and it also accounts for spatial heterogeneity of CBV in the brain. In this way, tumors localized to the cortex, where CBV is normally relatively high, would effectively have a higher threshold for defining abnormal tissue CBV compared with tumors localized to deep white matter regions, where CBV is relatively low. The atlas method to quantification of abnormal vasculature allows for better delineation of tumors that extend or infiltrate into multiple tissue types by using spatially dependent thresholds defined by the location on the atlas.

Results from the current study suggest the extent of hypervascular tumor as defined by comparison of individual patients to a population-based CBV atlas is a significant predictor of patient response and survival, whereas traditional measures of tumor perfusion such as mean CBV did not correlate with response or survival. Sawlani *et al.*[18], in a pilot study of 16 patients, measured the change in hyperperfusion volume (ΔHPV) due to treatment, where HPV was defined as the volume of relative cerebral blood volume >1.25. They found that this metric had a better correlation with progression-free survival than the more traditional CBV metrics. A more recent study by La Violette *et al*. [19] utilized a novel approach to post-processing of DSC-MRI to show that the volume of abnormal tumor vasculature could be used to predict response to bevacizumab in malignant gliomas. Together, these results appear to suggest that the volume of abnormal vasculature, as opposed to traditional perfusion MRI measures of CBV or CBF, many be more useful for monitoring the response to anti-angiogenic therapies.

There are a few study limitations that should be addressed. First, variations in scan parameters in bevacizumab-treated patients before and/or after therapy may have altered estimates of traditional CBV metrics. This bias is accounted for when using the atlas-defined perfusion parameters, since the atlas was constructed from DSC-MRI scans obtained under a variety of scan conditions (i.e. field strengths, temporal resolution, flip angles, etc.). Another potential limitation to the current study that should be addressed is the use of patients with brain tumors, not healthy control subjects, to populate the atlas. In the current study we chose to include only patients with brain tumors for two reasons: 1) An atlas composed of only brain tumor patients treated with a variety of therapies ensured that any increase in rCBV beyond these changes observed during therapies would be related to growing tumor and not due to a specific therapy. 2) Perfusion MRI with the use of contrast is only routinely obtained in patients with suspected or confirmed brain tumors, stroke, or other pathologies that may influence cerebral perfusion. Thus, building a large-scale database containing hundreds of age matched (i.e. older) healthy individuals with no suspected brain pathology is impractical (and some may argue could also be considered unethical). Additionally, patients were excluded from both construction of the atlas or assessment of anti-angiogenic therapy if DSC-MRI data had less than 6 slices, since this increased the difficulty of aligning it in standardized space and, in the case of treatment evaluation, rarely allowed for full coverage of the tumor.

## Conclusion

In the current study we have constructed a large-scale population image map of CBV from DSC-MRI data in 450 patients, then used this atlas to compare individual patient CBV maps with the purpose of defining the extent of abnormal tumor vasculature. Results suggest atlas-defined hypervascular tumor volumes predict both response and survival in recurrent glioblastoma patients treated with bavacizumab. This study highlights the importance of using large-scale population image maps to identify abnormal biological tissues.

## Competing interests

Drs. Timothy F. Cloughesy, Albert Lai, Whitney Pope, and Benjamin Ellingson are paid consultants for Genentech, Inc., and Hoffman-La Roche, Ltd. Drs. Ellingson and Pope are also a paid consultant for MedQIA, LLC.

## Authors’ contributions

KL constructed the radiographic atlas, contoured tumors, performed statistical analyses, and drafted the manuscript. DRE aided in study design, data interpretation, and manuscript editing. DCW aided in construction of the atlas, performed image registration, and edited the manuscript. RJH aided in construction of the atlas, performed image registration, and edited the manuscript. ANT aided in construction of the atlas, performed image registration, and edited the manuscript. AL aided in study design, gathering of clinical data, and manuscript editing. PLN aided in study design, gathering of clinical data, and manuscript editing. WBP aided in study design, provided radiologic expertise, provided clinical data, and aided with manuscript editing. TFC aided in study design, gathering of clinical data, data interpretation, and manuscript editing. BME designed the study, interpreted the data, created figures, provided statistical expertise, and was responsible the final edits. All authors read and approved the final manuscript.
